# Invasive Fungal Infections in Patients with Hematologic Malignancies (Aurora Project): Lights and Shadows During 18-Months Surveillance

**DOI:** 10.3390/ijms13010774

**Published:** 2012-01-13

**Authors:** Maria Teresa Montagna, Osvalda De Giglio, Christian Napoli, Grazia Lovero, Giuseppina Caggiano, Mario Delia, Domenico Pastore, Nicola Santoro, Giorgina Specchia

**Affiliations:** 1 Hygiene Section, Department of Biomedical Science and Human Oncology, University of Bari, Piazza G. Cesare 11, 70124 Bari, Italy; E-Mails: osvaldadegiglio@tiscali.it (O.D.G.); c.napoli@igiene.uniba.it (C.N.); raz.ro@tiscali.it (G.L.); caggianog@igiene.uniba.it (G.C.); 2 Hematology Section, Department of Pathological Anatomy, University of Bari, Piazza G. Cesare 11, 70124 Bari, Italy; E-Mails: mario.delia@tiscali.it (M.D.); d.pastore@ematba.uniba.it (D.P.); g.specchia@ematba.uniba.it (G.S.); 3 U.O. Pediatrics “F.Vecchio”, A.O.U. Policlinico Consorziale, Piazza G. Cesare 11, 70124 Bari, Italy; E-Mail: n.santoro@bioetaev.uniba.it

**Keywords:** fungal infections, onco-hematology units, surveillance

## Abstract

The aim of this multicenter prospective study was to evaluate the incidence of invasive fungal infections (IFIs) in adult and pediatric patients with hematologic malignancies, involving nine nosocomial facilities in Southern Italy over a period of 18 months. Furthermore, results of an environmental microbial surveillance routinely carried out in some of the enrolled hospitals are reported. A total of 589 onco-hematological patients were enrolled and 27 IFIs were documented. The main infections were caused by yeasts, more than filamentous fungi (overall incidence of 2.7% and 1.9%, respectively). The yeasts were mainly represented by *Candida* spp. (87.5%), all isolated by blood cultures; *C. parapsilosis* was the most common species. Among mould infections, the most frequent site was the lung, with regard to aspergillosis (81.8%). In six of the 10 patients with suspected aspergillosis, the diagnosis was made by the detection of galactomannan and (1,3)-β-d-glucan antigens. The microbiological surveillance carried out on 156 air, 312 water and 312 surface samples revealed low environmental contamination: *Alternaria alternata* was the only fungus isolated from two surface samples. Our data, especially the low occurrence of filamentous fungi, suggest a particular local epidemiology. Further studies are needed to confirm this microbiological trend in onco-hematological patients in Southern Italy, the results of which might be helpful to improve the management of these patients.

## 1. Introduction

The epidemiology of invasive fungal infections (IFIs) in onco-hematological patients has changed substantially in recent years and its incidence varies considerably among different nations [1–3]. A special vulnerability to these infections is introduced by risk factors related to the underlying disease (e.g., neutropenia especially for acute myeloid leukemia patients, steroids usage, high dose cytarabine, invasive medical devices) [4,5], genetic profile [6,7], pre-hospital and hospital exposure [8], age, and comorbidities.

Among IFIs caused by yeasts, *Candida albicans* remains an important pathogen, but a rising rate of infections is caused by non-*albicans Candida* species (*i.e.*, *C. glabrata*, *C. guilliermondii*, *C. parapsilosis*, *C. tropicalis* and *C. krusei*) [9–11].

Over the last 20 years, an increasing number of infections caused by moulds has been reported: *Aspergillus* spp. seems to be the main fatal complication in patients with hematological malignancies, but other opportunistic moulds, such as *Fusarium* spp. and *Zygomycetes*, have also been described [12], whereas infections caused by other filamentous fungi are still rare [13,14].

Although the actual incidence of the IFIs has increased, its real frequency is often underestimated because of the difficulty in diagnosis [15]. In fact, although the use of laboratory tests has expanded in recent years, IFIs diagnosis continue to be hampered by non-specific clinical manifestations and difficulties in obtaining appropriate biological samples for mycological investigations. Such investigations usually require invasive procedures (e.g., histological samples, bronchoalveolar lavage) often precluded by cytopenia or by the critical condition of these patients. Moreover, the newer diagnostic approaches, focusing on the detection of surrogate markers such us circulating fungal antigens or metabolites (e.g., galactomannan and (1,3)-β-d-glucan), need serum samples repeated over time because of their occasional presence in the blood [15].

This study is a subset of the AURORA Project, the first active surveillance program carried out in Apulia (Southern Italy) to assess the incidence of IFIs in patients admitted to the Intensive Care, Hematology and Neonatal Intensive Care Units. It was launched in February 2007 and approved by the Ethics Committee of the Azienda Ospedaliero-Universitaria Policlinico of Bari, Italy.

The present study reports the incidence of IFIs in onco-hematological patients, the characteristics of enrolled patients, and the role of the additional biomarkers-diagnostic tests. Moreover, the results from an environmental surveillance carried out on water, surface and air samples are reported.

## 2. Material and Methods

### 2.1. Study Design

Apulia is a region located in Southern Italy with about 4 million inhabitants, 38,000 births/year and a mean of 850,000 hospital admissions/year. All the regional hospitals admitting onco-hematological patients were involved in the AURORA project, for a total of nine nosocomial facilities: five admitting adult patients and four both adult and pediatric patients.

To standardize the clinical recruitment and microbiological procedures, a training course was organized before starting the study, and a manual was distributed to all participants.

The study was carried out over 18 months (February 2007–August 2008) on adult (aged over 16 years old) and pediatric (under 16 years old) patients with newly diagnosis of one of the following diseases:

- acute myeloid (AML) or acute lymphoid leukemia (ALL);
- chronic lymphocitic (CLL) or chronic myeloid leukemia (CML);
- Hodgkin’s (HL) or non-Hodgkin’s lymphoma (NHL);
- Multiple myeloma (MM)

Moreover, patients with autologous or allogeneic hematopoietic stem cell transplantation (HSCTs) following one of the previously mentioned onco-hematological malignancies were enrolled.

Patients with non-malignant hematological disorders (hemolytic anemia, thrombocytopenias, aplastic anemia, *etc*.) were excluded from the study.

In accordance with the criteria proposed by Ascioglu [16] and taking into account the clinical data of enrolled patients, biological samples from potentially infected sites (e.g., blood, peritoneal and cerebrospinal fluid, bronchial aspirate, sputum**)** were sent to each hospital-associated microbiology laboratory for the mycological analysis, where all fungal species were eventually isolated, identified and stored. Moreover, the circulating antigens (1,3)-β-d-glucan (BDG) and galactomannan (GM) were performed only in patients with suspected moulds disease (e.g., positive culture or instrumental tests, persisting fever, chest pain, *etc*.). Serum samples were analyzed thrice weekly until discharge or death.

For each enrolled patient, the participating centre had also to complete an electronic report form including information about the patient (age, sex, underlying disease), microbiological data (date of first culture, etiological agents, circulating antigens), histological and/or radiological evidence, therapeutic approach and outcome 30–60 days after enrolling.

In order to confirm the identification of species and to analyze the data, both the isolates and the electronic reports were sent to the Coordinating Centre (Laboratory of Mycology, Department of Biomedical Sciences and Human Oncology, University of Bari “Aldo Moro”).

As indicated in the study protocol, the pharmacological management of the onco-hematological adult patient was made according to ECIL 2007 [17] and IDSA 2008 [18] guidelines. In particular, prophylaxis was performed with Fluconazole (400 mg daily until recovery of neutrophils). In case of fever unresponsive to an adequate (at least 96 h) empiric broad spectrum antibiotic therapy, we proceeded with an empiric antifungal therapy using intravenous Caspofungin (loading dose of 70 mg, then 50 mg daily) or Lipid Formulation (LF) of Amphotericin B (3 mg/kg daily). Voriconazole (loading dose of 6 mg/kg × 2, then 3 mg/kg × 2 daily) or Caspofungin or LF Amphotericin B were also used in case of microbiological evidence coming from patients undergoing close surveillance (*i.e.*, serum detection of galactomannan antigen, detection of respiratory tract colonization by filamentous fungi and/or CT and ultrasonographic examination according to clinical features).

In the pediatric population the use of antifungal prophylaxis (Fluconazole at 6 mg/kg daily) was reserved for patients with severe neutropenia (PMN < 500/mm^3^), while an empiric antifungal therapy with Caspofungin (50 mg/m^2^ daily) or LF Amphotericin B (3 mg/kg daily) was administered in case of fever unresponsive to an adequate antibiotic therapy.

Furthermore, because an environmental microbial surveillance was carried out three times per year in the bone marrow transplant units located in 4 out of the 9 enrolled hospitals, a hypothetic association between the IFIs cases and the environmental fungal surveillance was evaluated. These wards are equipped with a laminar air-flow (LAF) system with HEPA filters. Each room has a positive airflow control device.

### 2.2. Case Definition

The diagnosis of invasive fungal disease was made according to the definition proposed by the EORTC/MSG [19]. Our analysis was restricted to infections classified as “proven*”* or “probable”.

The duration of fungemia was defined as the time interval (days) between the first and last positive blood cultures (yielding the same pathogen). Persistent fungemia was defined as the persistence of positive blood cultures for >2 days, from the time of the first positive blood culture.

### 2.3. Laboratory Procedures

Each biological sample (e.g., sputum, nasal swab) was cultured on two Sabouraud chloramphenicol dextrose agar (SDA) plates (bioMèrieux, Marcy l’Etoile-France), incubated at 36 ± 1 °C and 28 °C (for yeasts and moulds isolation) and examined daily for 10 days.

Identification of yeasts was performed with sugar assimilation profiles obtained using the ID32C and confirmed by VITEK-2 System (Biomérieux, Marcy l’Etoile, France).

Filamentous fungi were identified on the basis of their macroscopic and microscopic morphological features, in accordance with standard methods [20].

The presence of the BDG antigen was measured by colorimetric assay, Fungitell (Associates of Cape Cod Inc., E. Falmouth, MA, USA); the GM antigen was measured using a commercial sandwich immunoassay (Platelia *Aspergillus* Ag—BioRad, Marnes La Coquette, France). All tests were performed according to the manufacturer’s instructions.

### 2.4. Environmental Surveillance

Overall, 156 air, 312 water and 312 surface samples provided by the four hospitals including bone marrow transplant units (for a total of 26 onco-hematological patient’s rooms) were examined. SDA plates were employed to yield fungi, incubated at 36 ± 1 °C and 28 °C (for yeasts and moulds isolation) and examined daily for up to 10 days.

Air samples (180 L/min for a total suction volume of 500 L) were collected using a Surface Air System (SAS) sampler (Microflow Acquaria, Milan, Italy). It was placed in each monitored room about 1 m above the floor and 1 m from the patient’s bed. The number of colony forming units was adjusted using the conversion table provided by the manufacturer and was expressed in colony forming units per cubic meter (cfu/m^3^) [21].

For mycological water contamination, tap and shower water samples were collected in sterile bottles and immediately transported to the laboratory in cool boxes, in accordance with Italian legislation [22].

Fungal contamination of surfaces was evaluated at the patient’s bedhead and on the air aspiration grate, using sterile swabs [23]. The results were expressed as cfu/cm^2^.

### 2.5. Statistical Analysis

Data were entered into a computer database (Microsoft Access 2003). Categorical variables were expressed as proportions or percentages, and numerical data were expressed as the mean ± SD and range.

Univariate analysis was performed using the χ^2^ test and Fisher’s exact test where appropriate with each of the following independent variables: age group (0–16, 17–40, 41–60, 61–80), sex, underlying hematological diseases, host factors and autologous or allogeneic hematopoietic stem cell transplantation (HSCTs). Variables for which data sets were incomplete were not included. Multivariate analysis was performed using a logistic regression model applying the stepwise selection to identify significant associations between host factors and risk of developing an IFI. Adjusted odds’ ratio (OR) and 95% confidence interval (CI) were calculated. A *p* < 0.05 was considered statistically significant.

The analyses were performed using the software SAS system version 9.2.

The overall and IFI-attributable mortality rates (AMR) were estimated. As reported by Pagano *et al*. [14], we considered the “overall mortality” as the ratio of deaths affected by IFIs to the total number of enrolled patients and the AMR as the ratio of deaths in patients affected by IFIs to the number of enrolled patients affected by IFIs.

To evaluate the association between environmental fungal surveillance and presence/absence of IFIs, data regarding number of hospitals with and without a periodical environmental monitoring and number of hospitals with and without IFIs were entered in a contingence table. The relative risk (RR) and the confidence interval at 95% were calculated. The *Chi-square* test was applied to evaluate the significance of association between variables, considering significant a *p*-value < 0.05.

## 3. Results

During the 18-months study, 589 patients with newly diagnosed hematological malignancies were enrolled (475 adults and 114 pediatrics). Their underlying hematological diseases and host factors are shown in Table 1.

Twenty-seven episodes of IFIs were documented: overall incidence 4.6% (5% in adults and 2.6% in pediatric patients). The male/female ratio was 13/11 in the adult population (mean age at diagnosis = 53.6 ± 16.6 years; range, 19–78) and 2/1 in the pediatric population (mean age at diagnosis = 6 ± 6.1 years; range, 2–13). The most common underlying disease was AML (40.7%), followed by NHL (18.5%), ALL (14.8%), CLL and MM (11.1%, respectively) and HD (3.7%).

Of the 27 documented IFIs, 16 were caused by yeasts (13 in adult patients: incidence 2.7% and 3 in pediatric patients: incidence 2.6%) and 11 by filamentous fungi (all in adult patients: incidence 2.3%). No filamentous fungi infections in pediatric patients were reported.

Table 2 shows the IFIs incidence by yeasts and moulds distributed for different types of hematologic malignancies, host factors and hematopoietic stem cell transplantation (autologous and allogeneic bone marrow transplant). In adult patients, the incidence ranged between 1.4% among patients with NHL and 4.1% in those with AML for yeasts infections; between 1.4% among patients with NHL and MM and 30% in those with CLL for moulds infections. Pediatric patients developed only yeasts infections, whose incidence was 14.3% among patients with NHL and 2.3% in those with ALL. Of the 27 patients, 5 underwent hematopoietic stem cell transplantation: three autologous and two allogeneic bone marrow transplants. The three patients with autologous bone marrow transplant (2 MM and 1 NHL) had only yeasts IFIs; the two patients with allogeneic bone marrow transplant (both AML) had one yeast and one mould IFI respectively.

Regarding the antifungal treatment, 67% of our patients were undergoing empiric therapy. The overall fungal infections mortality rate was 1% (6/589 patients) and the AMR was 22.2% (6/27 cases). Fisher’s exact test showed a significant difference between AMR for mould *vs.* yeast infections (45.4% *vs.* 6.25%; *p* = 0.026); it must be considered that no pediatric patient died.

### 3.1. Yeast Infections

Of the 27 diagnosed cases, 16 (59.2%) were caused by yeasts, all based on blood cultures: 13 were adult (male/female ratio was 8/5) and 3 pediatric patients (male/female ratio was 2/1). The majority of yeasts isolates were *Candida* spp. (87.5%); *C. parapsilosis* was the most frequently isolated species in adult (36.4%), followed by *C. tropicalis*, C. *krusei*, *C. glabrata* (18.2% respectively) and *C. guilliermondii* (9%). *Cryptococcus neoformans* and *Geotrichum capitatum* (both 6.2%) were also isolated.

With regard to adult patients, the underlying diseases were AML (61.5%), NHL (15.4%), MM (15.4%) and ALL (7.7%). Among the host factors, the presence of a central venous catheter (CVC) resulted the most frequent (69.2%), followed by chemotherapy (cytarabine in all cases) (53.8%), severe neutropenia (38.5%, with a mean duration of 7 ± 1.6 days; range 6–11), prolonged antibiotic therapy (30.8%), and immunosuppressive therapy (15.4%) with cyclosporine. Moreover, four were bone marrow transplant patients: autologous transplants (23.1%) were more frequent than allogeneic ones (7.7%). The mean age of adult patients at diagnosis was 55.1 ± 14.6 years (range 19–77). The time of infection onset was 15 ± 16.1 days (range 0–55) from the first day of recovery; the mean duration of fungemia was 3.8 ± 6.3 days (range 1–24); persistent fungemia was found in four patients (30.8%—range, 4–24 days). Just one adult patient with yeast infection and MM who underwent autologous bone marrow transplant died. In five adults, at the moment of the IFI suspect and after the blood sampling, the CVC was removed and underwent catheter tip cultures. In all cases, the blood culture tested positive for *Candida parapsilosis*; moreover, three CVC resulted positive for the same fungal species (*C. parapsilosis*) and two were negative.

With regard to pediatric patients with yeasts IFI, the underlying diseases were ALL (2/3: 66.6%) and NHL (1/3: 33.3%). They had a central venous catheter and were under chemotherapy, immunosuppressive therapy, and use of corticosteroids; moreover, 66.6% of them showed severe neutropenia. The mean age of pediatric patients at diagnosis was 6 ± 6.1 years (range 2–13), the mean duration of fungemia was 4 ± 3 days (range 1–7); persistent fungemia was found in two patients (4 and 7 days, respectively). The time of onset of infection from the first day of recovery was 19.3 ± 19.6 days (range 1–40), caused by *C. albicans*, *C. lusitaniae* and *C. parapsilosis* (33.3% respectively). The three children with IFI did not have the CVC removed.

Univariate analysis showed that—in adult patients—chemotherapy (*p* < 0.0001), autologous bone marrow transplant (*p* < 0.05) and severe neutropenia (*p* < 0.05) were significantly associated with yeasts IFIs. Moreover, the stepwise regression analysis did not show any significant risk factor for developing yeasts IFIs. With regard to yeasts infections in children, univariate and multivariate analysis did not show any risk factors for developing IFI.

### 3.2. Mould Infections

A total of eleven mould infections (40.7%, 10 aspergillosis and 1 zygomycoses) were reported only in adult patients (incidence 2.3%). The overall male/female ratio was 5/6. The mean age at diagnosis was 51.8 ± 19.2 years (range 21–78).

Underlying diseases were AML and CLL (27.3%, respectively), NHL (18.2%), HD, ALL and MM (9.1%, respectively).

Severe neutropenia (<500 PMN/mm^3^) resulted the most frequent host factor (81.8%), followed by central venous catheter (45.5%), corticosteroids therapy and chemotherapy (36.4%, respectively), prolonged antibiotic therapy and *Cytomegalovirus* infection (18.2%, respectively).

The most common site of infection was the lung, with regard to aspergillosis (81.8%). These patients presented a CT scan suggestive of moulds infection, but only in three cases the etiological agents were isolated from respiratory secretions and identified at the species level (*A. fumigatus*). The remaining two patients were positive in different sites, one for *Aspergillus fumigatus* recovered by two blood cultures and one for *Rhizomucor pusillus* by a retrosternal lesion.

BDG, GM antigens detection was performed in all eleven patients, for a total of 39 sera. BDG tested positive (>80 pg/mL) in all the 38 sera samples from patients with aspergillosis (10/11), while GM antigen tested positive (Index cut-off > 0.5) in 36 of the same 38 sera samples. Using BDG and GM assays, an aspergillosis diagnosis was possible in 6 of the 10 patients with suspected aspergillosis, in which the culture survey was negative.

The AMR in adult patients was estimated at 45.4% (5/11 cases): one AML patient had undergone an allogeneic bone marrow transplant (9.1%), two patients were affected by NHL (18.2%) and two by CLL (18.2%).

Univariate analysis showed that chemotherapy (*p* < 0.0001) and *Cytomegalovirus* infections (*p* < 0.01), were significantly associated with mould IFIs in adult patients. Moreover, the stepwise regression analysis confirmed only *Cytomegalovirus* infection (*p* = 0.0012; OR 15.419; CI 95% 1.814–131.103) to be a risk factor for developing mould IFIs.

With regard to the pediatric patients, no mould IFIs were detected.

### 3.3. Environmental Surveillance

In four Hematology units, including bone marrow transplant wards, equipped with HEPA filters and undergoing environmental monitoring, fungi were never isolated from air/water samples collected from patients’ rooms; only *Alternaria alternata* was isolated from two surface samples collected at the same time from a room. In these hospitals a single case of mould IFI was diagnosed.

In the remaining five hospitals without HEPA filters and not undergoing environmental monitoring, 10 mould IFIs were detected.

Since this surveillance yielded a small number of IFIs, an association between the results of environmental surveillance and frequency of IFIs was not possible.

## 4. Discussion

In Apulia region the IFIs affecting onco-hematological patients appear to be caused more commonly by yeasts than by filamentous fungi (59.2% *vs.* 40.7%), with an overall incidence of 2.7% and 1.9% respectively.

Among yeasts, *Candida* non *albicans* (CnA) species were the most frequently isolated, according to a retrospective study carried out during 1998–2004 in one of the hospitals enrolled in the present survey [24]. These data revealed an evident increase of CnA candidemia, particularly *C. parapsilosis* in Hematology and Pediatric Oncology Units. By contrast, *C. albicans* was the major cause of candidemia in the other wards. Also in our study *C. parapsilosis* resulted in the most frequent CnA species (38.5%). Although this yeast is one of the less virulent species of *Candida*, its role in epidemiology of blood stream infections is very important because it is often responsible for healthcare-associated infections (e.g., intravascular catheters and parenteral nutrition) [25].

Other yeasts isolated from blood were *Cryptococcus neoformans* and *Geotrichum capitatum* (6.2% both) in two different AML patients. These data are in accordance with other reports that show a lower incidence of blood stream infections caused by these yeasts in patients with hematologic malignancy [13,14].

Regarding mould infections, we found a low incidence in adult patients (11/475; 2.3%) and no case in the 114 pediatric patients. We realize that these data are not according to those reported by other authors. In fact, recent studies indicate that:

- AML patients are at highest risk of developing an IFI [14,26]. Our data confirm these findings only with regard to yeast infections. Paradoxically, the incidence of mould infections was higher in patients with CLL (30%) than in those with AML (1.5%, one proven zygomycosis and two probable aspergillosis), probably because of the wide use of monoclonal antibodies therapy (Alemtuzumab), that the CLL patients receive for 18 weeks. In fact, in a recent study some authors [27] state that the incidence of moulds IFIs increases after this kind of immunosuppressive therapy.
- Besides *Aspergillus* spp., other moulds (*i.e.*, *Zygomycetes*, *Fusarium*, *Scedosporium*) are emerging, probably related to the use of more aggressive chemotherapy [14]. In our survey, only one out of 11 mould infections was caused by *Zygomycetes*, in a patient undergoing more than three lines of chemotherapy.
- Pagano *et al*. [27] report results “enthusiastic” in the outcome of mould infections, particularly for aspergillosis. Our AMR for moulds is higher than that found for yeasts (45.4% *vs.* 6.2%; *p* = 0.026), probably due to late diagnosis because of non-specific clinical signs or to a poor sensitivity of conventional cultural methods. Alternative approaches, like the search for circulating antigens (BDG, GM), could represent a new way to achieve an early and valid diagnosis, but these methods are not accessible to all laboratories (e.g., due to high cost). For this reason, BDG and GM were performed only in patients with suspected moulds disease, resulting in the diagnosis of six cases of probable aspergillosis always with negative cultures [19].

It is possible that our data concerning the low incidence of IFI from moulds may also be related to empirical antifungal therapy administered in 67% of enrolled patients, or to preventive measures (e.g., ventilation system cleaning and HEPA-LAF filtered room) usually adopted in four out of the nine enrolled hospitals. In fact, in these wards, a microbiological surveillance is regularly carried out on air, water and surfaces three times per year. In our 18 months experience, this surveillance yielded a poor environmental contamination: the only filamentous fungus detected was *Alternaria alternata* on a table surface, never identified as etiological agent of fungal disease. These results are in accordance with those of a previous environmental surveillance carried out in the operating theatre of one of the hospitals enrolled in the Aurora Project [28]. Also other authors [29,30] reported that air contamination was never detected in/outside the flow of HEPA-LAF rooms. So, the environmental protective facilities (air filtration and/or laminar airflow) could be of benefit for these high-risk patients, especially during treatment periods [31].

There are other potential reasons explaining the low incidence of moulds IFI: a different geographic distribution of some fungal species [2,32], the absence of restructuration works in most enrolled hospitals during the surveillance period, the meteorological conditions (e.g., low humidity and rainfall in winter, very hot summer), all factors promoting or limiting the environmental spread of fungal spores.

Regarding bone marrow transplant patients, only three yeasts IFIs were reported in adults. Our data are in agreement with Pagano *et al*. [3] who showed that yeasts IFIs were more frequent than moulds diseases in recipients of HSCT.

Regarding pediatric patients, the yeasts IFIs incidence was 2.6% (2 ALL and 1 NHL patients); these data support findings from Castagnola *et al*. [33] that found an incidence of 1.8%. About mould infections, no cases were detected in our pediatric patients. Zaoutis *et al*. [34], during a study of invasive aspergillosis (IA), reported the highest incidence in allogeneic bone marrow transplanted children (4.5%) and with acute myeloid leukemia (4%), but our enrolled children were mostly affected by ALL (77.2%) and had no transplantations. We reported no deaths for IFI in these patients, according to Kaya *et al*. [35] who described just one death among 21 pediatric leukemia patients with IFIs.

## 5. Conclusions

Our conflicting results with regard to the numbers and type of fungal infections might be mainly explained by the high rate (67%) of empirical antifungal therapy which usually results in a lower rate of proven/probable IFI, because of a less intense diagnostic work-up. Actually, BDG and GM determinations were performed only in patients with suspected infections as well as TC thorax scan was not regularly carried out and consequently BAL was exceptional. Although the diagnostic driven/pre-emptive approach was to prefer in Aurora Project experience because of the patients’ various fungal risk, the empirical approach was the prevalent one and the IFI rate inevitably penalized. Any effort should be made in order to facilitate the implementation of diagnostic microbiological test (*i.e.*, BDG and GM) as well as radiological examinations in hematological patients’ IFI work-up.

## Figures and Tables

**Table 1:** Demographics and clinical characteristics of 589 enrolled patients.

Characteristics	All patients (*n* = 589) No. (%)	Adults (*n* = 475) No. (%)	Pediatric (*n* = 114) No. (%)
Sex			

Male	383 (65)	290 (61)	93 (81.6)
Female	206 (35)	185 (38.9)	21 (18.4)

Age-mean ± SD (years)	47.7 ± 23.4	57.3 ± 13.7	7.4 ± 5.7
[range]	[2–80]	[19–80]	[2–16]
0–16	114 (19.3)	—	114 (100)
17–40	14 (2.4)	14 (2.9)	—
41–60	200 (33.9)	200 (42.1)	—
61–80	261 (44.3)	261 (54.9)	—

Underlying hematological diseases			

AML	204 (34.6)	195 (41)	9 (7.9)
ALL	136 (23.1)	48 (10.1)	88 (77.2)
CML	5 (0.8)	5 (1.0)	0
CLL	10 (1.7)	10 (2.1)	0
NHL	147 (24.9)	140 (29.7)	7 (6.1)
HD	15 (2.5)	5 (1.0)	10 (8.8)
MM	72 (12.2)	72 (15.1)	0

Host factors			

Chemotherapy	580 (98.5)	466 (98.1)	114 (100)
Central venous catheter	343 (58.2)	238 (50.1)	105 (92.1)
Prolonged antibiotic therapy	223 (37.9)	143 (30.1)	80 (70.2)
Neutropenia < 500 PMN/mm^3^	410 (69.6)	333 (70.1)	77 (67.5)
Neutropenia: 500–1000 PMN/mm^3^	179 (30.4)	142 (29.9)	37 (32.4)
Immunosuppressive therapy	138 (23.4)	24 (5.0)	114 (100)
Corticosteroids	209 (35.5)	95 (20)	114 (100)
Parenteral nutrition	101 (17.1)	95 (20)	6 (5.3)
Surgery	25 (4.2)	5 (1)	20 (17.5)
*Cytomegalovirus* infection	12 (2)	12 (2.5)	0

HSCT			

Allogeneic bone marrow transplant	15 (2.5)	15 (3.1)	0
Autologous bone marrow transplant	30 (5.1)	30 (8)	0

AML: acute myeloid leukemia; ALL: acute lymphoid leukemia; CML: chronic myeloid leukemia; CLL: chronic lymphocitic leukemia; NHL: non-Hodgkin’s lymphoma; HD: Hodgkin’s disease; MM: multiple myeloma; HSCT: hematopoietic stem cell transplantation.

**Table 2:** Incidence of invasive fungal infections (IFIs) in adult and pediatric patients.

Hematologic malignancies	Adult patients		Pediatric patients

	Yeasts IFI No./Total (Incidence %)	Moulds IFI No./Total (Incidence %)	Yeasts IFI No./Total (Incidence %)
AML	8/195 (4.1)	3/195 (1.5)	0
ALL	1/48 (2.1)	1/48 (2.1)	2/88 (2.3)
CLL	0	3/10 (30)	-
NHL	2/140 (1.4)	2/140 (1.4)	1/7 (14.3)
HD	0	1/5 (20)	0
MM	2/72 (2.8)	1/72 (1.4)	-

**Host factors**			

Chemotherapy	7/466 (1.5)	5/466 (1.1)	3/114 (2.6)
Central venous catheter	9/238 (3.8)	5/238 (2.1)	3/105 (2.8)
Prolonged antibiotic therapy	4/143 (2.8)	2/143 (1.4)	0
Neutropenia <500 PMN/mm^3^	5/333 (1.5)	9/333 (2.7)	2/77 (2.6)
Neutropenia: 500–1000 PMN/mm^3^	1/142 (0.7)	1/142 (0.7)	1/37 (2.7)
Immunosuppressive therapy	2/24 (8.3)	1/24 (4.2)	3/114 (2.6)
Corticosteroids	0	4/95 (4.2)	3/114 (2.6)
Parenteral nutrition	4 (4.2)	0	0
Surgery	1 (20)	0	0
*Cytomegalovirus* infection	0	2/12 (16.7)	0

**HSCT**			

Allogeneic bone marrow transplant	1/15 (6.7)	1/15 (6.7)	-
Autologous bone marrow transplant	3/30 (10)	0	-

AML: acute myeloid leukemia; ALL: acute lymphoid leukemia; CLL: chronic lymphocitic leukemia; NHL: non-Hodgkin’s lymphoma; HD: Hodgkin’s disease; MM: multiple myeloma; HSCT: hematopoietic stem cell transplantation.
